# Relation of Asymmetric Dimethylarginine Levels to Macrovascular Disease and Inflammation Markers in Type 2 Diabetic Patients

**DOI:** 10.1155/2014/139215

**Published:** 2014-04-03

**Authors:** Mustafa Celik, Serkan Cerrah, Mahmut Arabul, Aysen Akalin

**Affiliations:** ^1^Department of Gastroenterology, Izmir Ataturk Training and Education Hospital, Katip Celebi University, Turkey; ^2^Department of Gastroenterology, Erzurum Regional Research and Hospital, Turkey; ^3^Department of Endocrinology, Eskisehir Osmangazi University Medical School, Eskisehir, Turkey

## Abstract

*Aim*. We aimed to determine the relation of asymmetric dimethyl arginine (ADMA) levels to atherosclerotic vascular disease and inflammation markers in type 2 diabetes. *Methods*. We recruited 50 type 2 diabetic patients with atherosclerosis, 50 type 2 diabetic patients without atherosclerosis, and 31 healthy control patients into our study. We obtained fasting serum and plasma samples and measured HbA1c, fasting blood glucose, C-peptide, creatinine, total cholesterol, triglycerides, HDL cholesterol, LDL cholesterol, hsCRP, fibrinogen, erythrocyte sedimentation rate, total homocysteine, and ADMA levels. In addition, all of the patients were evaluated for carotid artery intima media thickness by ultrasound. We evaluated ADMA levels in healthy controls, diabetic patients with macrovascular complications, and diabetic patients without macrovascular complications and evaluated the relationship between ADMA levels and total homocysteine, inflammation markers, and macrovascular disease. *Results*. Mean ADMA values in non-MVD and control groups were significantly lower than in MVD group (0.39 ± 0.16, 0.32 ± 0.13, 0.52 ± 0.23, *P* < 0.05, resp.). These three variables (carotid intima-media thickness, inflammatory markers, and ADMA levels) were significantly higher in diabetes group than control (*P* < 0.05). *Conclusion*. There is a relationship between ADMA and macrovascular disease in type 2 diabetes, but further studies are needed to understand whether increased ADMA levels are a cause of macrovascular disease or a result of macrovascular disease.

## 1. Introduction

Type 2 diabetes mellitus (DM) is one of the underlying causes of patient morbidity and mortality and is an important risk factor for coronary artery disease development. Compared to healthy populations, type 2 diabetic patients have a 2-3 times higher risk of macrovascular disease [1]. Coronary artery disease resulting from diabetes is responsible for 75% of diabetes related deaths [2].

Mechanical or functional damage of the endothelium results in diminished blood vessel function and can initiate the development of atherosclerosis. Studies have shown that the presence of endothelial dysfunction is an important prognostic indicator for future cardiovascular events [3, 4]. Endothelial dysfunction, on the other hand, can also result from decreases in free radicals, which are derived from oxygen and/or endothelial nitric oxide synthase (eNOS) activity and/or expression.

Asymmetric dimethylarginine (ADMA), an NOS inhibitor, is considered an indicator of endothelial dysfunction due to increased expression of ADMA in renal failure, coronary artery disease, apoplexy, hypertension, and diabetes mellitus [5, 6].

The idea that ADMA levels may not only be an indicator of endothelial dysfunction but may also play a role in increasing endothelial dysfunction demonstrates the need for further research. Atherosclerosis is considered an inflammatory process and increases in inflammation markers reflect the atherosclerotic process. These studies suggest that more information could be gathered and new treatment modalities could be developed in the prevention of endothelial dysfunction and atherosclerosis.

In this study, we examined the relationship between the ADMA levels in type 2 DM patients and indicators of macrovascular disease and inflammation.

## 2. Materials and Methods

Out of the 131 patients recruited into the study, 100 were diagnosed with type 2 DM, half of which also had atherosclerotic vascular disease. The remaining 31 patients were healthy. The research was reviewed and approved by the Eskişehir Osmangazi University Ethics Committee.

Patients with a history of myocardial infarction, coronary artery bypass operation, peripheral artery disease, and an apoplexy incident, ECG findings that hinted angina pectoris or ischemia or angiographically proven coronary artery disease were included in the macrovascular disease (MVD) group. Type 2 diabetic patients who did not have any known myocardial infarction, coronary artery bypass operation, peripheral artery disease, apoplexy, or angiographically proven coronary artery disease were included in non-MVD group. Patients with no evidence of pathology were included in the control group after receiving a verbal record of their mental history and a physical examination. Age, sex, BP levels, duration of diabetes, types of treatment, additional diseases of the patients,and the medicine prescribed were all noted.

Preprandial serum and plasma samples obtained from patients were tested for HgA1c, glucose, C-Peptide, total cholesterol, LDL, creatinine, hsCRP, fibrinogen, and erythrocyte sedimentation rate. Patients with hypothyroidism, vitamin B12 or folic acid deficiency after thyroid function tests were excluded from the study. The patients with creatinine levels above 1.8 mg/dL were not included in the study to eliminate any possible effects of their uremia. Patients recently diagnosed with diseases that could affect inflammation parameters, such as diabetic foot infection, were also excluded from the study.

Preprandial serum samples were kept at −70°C until total homocysteine (tHcy) (using Immulite 2000 Homocysteine kit, Siemens Medical Solutions Diagnostic, USA, and Competitive Immunoassay method) and ADMA (using ADMA direct ELISA Kit, Immunodiagnostic AG, Bensheim, Germany) were measured. Carotid artery intima media thickness was ultrasonographically evaluated.

The correlation between ADMA levels, tHcy, inflammatory markers and MVD was determined in the healthy control group, in diabetic patients with MVD and in diabetic patients non-MVD. 

*Statistical Analysis.* Descriptive statistics of the data were summarized as mean ± standard deviation. The Shapiro-Wilk test was used to determine whether variables were normally distributed. Parametric tests were conducted on normally distributed variables and nonparametric tests on variables with nonnormal distributions. Student's *t*-test and Mann-Whitney *U* test were used to compare two independent groups' means and medians, respectively. One-way ANOVA was used for comparison of three groups' means and Kruskal-Wallis test was used for differences among the groups' medians. The relationships between two categorical variables were evaluated by using chi-square test. Correlation analysis was used for the relation between numerical variables in each group. The linear relationships between ADMA and risk factors were evaluated by using multiple linear regression models. *P* < 0.005 was considered statistically significant. SPSS for Windows 15.0 was used for statistical analyses.

## 3. Results

Fifty patients with MVD and 50 patients with non-MVD, comprising the 100 type 2 diabetic patients in addition to 31 healthy control patients, were recruited for the study. The relationships of the variables age, body mass index (BMI), systolic blood pressure (SBP), diastolic blood pressure (DBP), duration of diabetes (D. of Diabetes), HbA1c, CrCl, albuminuria, PBG, c-peptide, creatinine, triglyceride, total cholesterol, HDL, LDL, sedimentation, hsCRP, fibrinogen, tHcy, vitamin B12, folic acid, right carotid intima media thickness (Car.Int-R), left carotid intima media thickness (Car.Int-L), ADMA to one another in MVD, non-MVD, and the control group, respectively, were evaluated by one-way ANOVA and Kruskal-Wallis tests (Table 1). The relationships of the common variables to one another in diabetic and healthy patients were also evaluated by *t*-test and Mann-Whitney *U* test (Table 2).

Sedimentation, CRP, and fibrinogen levels were measured as markers of inflammation and groups with respect to these three markers were compared. Mean of these 3 markers in MVD group compared to the control group were higher, but the differences between MVD and non-MVD, non-MVD, and control groups were not found significantly.

Mean ADMA value in non-MVD and control groups were significantly lower than in MVD group, respectively (*P* < 0.05 for each). According to these results, ADMA may be associated with macrovascular complications. In terms of levels of ADMA, there was no significant difference between the non-MVD and control groups.

The mean value of Car.Int-R variable in MVD group was found to be higher than control group (*P* < 0.05). In addition the mean of MVD and non-MVD groups were found to be higher than control group with regard to Car.Int-L.

The data were divided into a control group and patients group with all diabetes, and these two groups were compared in terms of common measurements. The results obtained are given in Table 2. There was no significant difference between the two groups in terms of HDL but triglyceride levels were higher in diabetic group than the control group. Cholesterol levels and LDL cholesterol levels in the control group were significantly higher than those of diabetes (*P* values < 0.05 and < 0.01, resp.). This result is connected to diet factor and statin use of the diabetic patients.

The mean Car.Int-R and Car.Int-L values, which were recognized as one of the important markers of atherosclerosis, of patients with diabetes are significantly higher than the control group (*P* < 0.01, for each). This result showed once again that diabetes is a major risk factor for atherosclerosis.

Sedimentation, hsCRP and fibrinogen levels were measured as markers of inflammation. Two groups compared with each other about these three markers, means of these 3 markers in diabetes group were found higher than control group (*P* < 0.01 for each). In addition we showed that the mean ADMA value of diabetic group were significantly higher than the control group's (*P* < 0.001).

As a result, because “These three variables (carotid intima-media thickness, inflammatory markers, ADMA levels) were significantly higher in diabetes group than control (*P* < 0.05)”, while in that “Because these three variables (carotid intima-media thickness, inflammatory markers, ADMA levels) were significantly higher than diabetes group than control (*P* < 0.05)”.

In the first two tables, inflammatory markers, creatinine, and ADMA levels which tended to increase with macrovascular disease and with diabetes are shown.

Later in the study, the correlations between ADMA levels and creatinine, inflammatory markers (sedimentation, hsCRP, and fibrinogen), and tHcy levels in all groups were evaluated. The results obtained are presented in Table 3.

ADMA and creatinine levels were positively correlated (*P* < 0.05) (Table 3). But there were no significant relationships between ADMA and sedimentation, hsCRP, fibrinogen, and tHcy.

In the MVD group, the relationships between ADMA and creatinine levels, sedimentation, hsCRP, fibrinogen, and tHcy were not found significant (Table 4).

In addition ADMA was evaluated by multiple regression analysis in which ADMA was taken as a dependent variable and T. cholesterol, PBG, C peptide, duration of diabetes, age, sedimentation, Car.Int-R, Car.Int-L, tHcy, hsCRP, BMI, HDL, HgA1c, CrCl, creatinine, albuminuria, fibrinogen, triglyceride, and LDL were taken as independent variables in MVD group. As a result, we showed that tHcy levels had significant effects on ADMA in Table 5. The relation between tHcy levels and ADMA was positive.

## 4. Discussion

Type 2 diabetes mellitus is one of the main causes of morbidity and mortality in the world, and its prevalence has increased dramatically in the past ten years [7]. Atherosclerosis is one of the underlying causes of morbidity and mortality in patients with type 2 DM, and type 2 diabetic patients are 2-3 times more likely to develop macrovascular disease [1].

Late complications from microangiopathy and macroangiopathy are burdens on both patient health and the economy. In treating and preventing type 2 DM the principal aim should be not merely to decrease the incidence of metabolic disease but also to minimize microvascular and macrovascular complications [8].

Patients at risk for atherosclerosis often have endothelial dysfunction. The relationship between oxidative stress, which increased by the NOS inhibitor ADMA, and endothelial dysfunction has been studied in many experimental animal models and diseases. Many studies have shown that increased ADMA in endothelial dysfunction is related to an increase in the generation of reactive oxygen species in plasma [8–10]. It has also been shown that endothelial dysfunction in coronary and peripheral vascular disease is connected with increased plasma ADMA concentration [11].

Studies have shown that ADMA levels increase in patients with cardiovascular risk [12, 13]. Macrovascular disease is 2-3 times more common in type 2 diabetics compared to normal population [1]. Krzyzanowska and colleagues showed that ADMA is related to clinical macrovascular atherosclerotic disease diagnosis in type 2 diabetics [14]. Research has also shown that increased tHcy concentration in type 2 diabetics is associated with increased cardiovascular disease [15, 16]. In our study, the ADMA level was found to be higher in diabetic patients with macrovascular complications compared to diabetic patients without complications. In addition, when all diabetic patients were compared to the control group, the ADMA level was higher in the diabetic group. We hypothesized that ADMA might be related to macrovascular atherosclerotic disease in type 2 diabetics. Moreover Krzyzanowska et al. concluded that ADMA is associated with tHcy, albuminuria, creatinine, and GFR and that tHcy correlates with age, ADMA, creatinine, GFR, LDL, and DTA [14]. In our study, we found a positive correlation between ADMA and age in diabetic patients with macrovascular complications and also found a correlation between ADMA and creatinine in all patients. No statistically significant relation between ADMA and tHcy was found in our study. Additionally, using ADMA as a dependent variable in multiple regression analysis, the levels of fundamental determinants of ADMA were evaluated in diabetic patients with macrovascular complications. It was found that ADMA's most fundamental determinant is tHcy. The data showed that ADMA is increased during atherosclerosis and DM, that ADMA is involved in the development of atherosclerosis, and that the fundamental determinant of ADMA is tHcy. Thus, ADMA may be used to predict the likelihood of developing macrovascular disease in diabetic patients.

Correlations between the levels of ADMA [17] and tHcy [18] and increased carotid intimamedia thickness during atherosclerosis development have been shown. In our study, ADMA levels were found to be much higher in diabetic patients with macrovascular complications than in the control group. When all diabetics were compared to the control group, the ADMA level in the diabetic group was found to be significantly higher. Also, carotid intimamedia thickness values in diabetic patients with macrovascular complication were higher than in the control group. When all diabetics were compared to the control group, carotid intimamedia thickness values in all diabetics were found to be higher than in the control group. However, using correlation analysis, no significant relation between ADMA and carotid intimamedia thickness was found. In addition, there was no significant difference in tHcy levels between groups. However, when all diabetics were considered there was a positive correlation between tHcy and carotid intimamedia thickness. The fact that ADMA levels in diabetic patients with macrovascular complications were higher made us question whether ADMA correlates with atherosclerotic development in the control group. In addition, since ADMA in diabetic patients was higher compared to the control group, we hypothesized that ADMA might have a role in atherosclerotic development in patients with DM, which is one of the main risk factors of atherosclerosis.

Various studies have shown that the inflammatory factor CRP level in type 2 diabetics is indicative of cardiovascular risk [19–21]. Moreover, Krzyzanowska and colleagues showed that ADMA is a new indicator of cardiovascular risk in type 2 diabetics and that it, independently of other known risk factors, increases the predictive value of CRP for cardiovascular disease [22]. Yet another study found that ADMA correlates with macrovascular disease and subclinical inflammation in type 2 diabetics [23]. In our study, inflammatory markers hsCRP, sedimentation, and fibrinogen were measured. All three inflammatory factors were significantly higher in diabetic patients with macrovascular complication than in the control group. Also, when all the diabetics were compared with the control group, the levels of the inflammatory markers were dramatically higher in the diabetic patients. However there was no significant correlation between ADMA and the inflammatory indicators. Corresponding to the existing evidence in diabetics, ADMA might be an indicator of macrovascular disease independent from inflammatory markers.

Consequentially, it is accepted that ADMA levels, a NOS inhibitor, can be used to diagnose macrovascular disease and cardiovascular mortality risk in type 2 diabetics. In our study, we concluded that ADMA's significance in the diagnosis of macrovascular disease risk is independent from other inflammatory markers. Thus, ADMA should be considered independently of the inflammatory markers in the prediction of macrovascular disease. Nevertheless it is not clear whether ADMA is a result of macrovascular disease development or a causative factor in macrovascular disease development. For this reason additional studies are needed to elucidate the effects of treatments targeted at decreasing ADMA levels and thus decreasing macrovascular disease development. 

*Results*

1. ADMA levels in diabetic patients with macrovascular complications were significantly higher than in patients without macrovascular complications and the control group (*P* < 0.05). Thus, ADMA might be related to macrovascular complication development.
2. When all the diabetics were compared to the control group, ADMA levels in the diabetics were significantly increased (*P* < 0.001). It was shown that ADMA tends to increase in DM, which is a main cause of atherosclerosis.
3. Sedimentation, hsCRP, and fibrinogen levels, which are inflammatory markers, were significantly higher in diabetic patients with macrovascular complications than in the control group (*P* < 0.05).
4. When all the diabetics were compared to the control group, the inflammatory markers sedimentation, hsCRP, and fibrinogen levels were significantly higher compared to the control group (*P* < 0.01). It was shown that inflammatory markers might be indicative of macrovascular complications and also that these markers increase in DM.
5. No significant difference in tHcy levels was found between the groups.
6. No significant correlation was found between ADMA and inflammation markers or homocysteine. This suggests that ADMA might be causing macrovascular complications independently of homocysteine and the inflammatory markers.
7. The levels of fundamental determinants of ADMA were evaluated in diabetic patients with macrovascular complications using multiple regression analysis in which ADMA was the dependent variable. We found that ADMA's most fundamental determinant is homocysteine.

## Figures and Tables

**Table 1:** Comparison of the common parameters in MVD, non-MVD, and the control group.

	MVD (a)	non-MVD (b)	Control (c)	
Age (years)	61.86 ± 7.96	56.8 ± 8.94	55.06 ± 7.46	(a-c) *P* < 0.001, (a-b) *P* < 0.01
BMI (kg/m^2^)	29.59 ± 4.76	30.44 ± 6.22	27.83 ± 2.68	*P* > 0.05
SBP (mm/Hg)	126.20 ± 13.07	125.40 ± 15.31	121.77 ± 9.53	*P* > 0.05
DBP (mm/Hg)	80.80 ± 8.16	77.90 ± 12.86	80.80 ± 8.76	*P* > 0.05
D. of diabetes (Years)	12.98 ± 7.24	9.08 ± 5.75		*P* < 0.01
HbA1c (%)	9.41 ± 2.63	9.88 ± 2.93		*P* > 0.05
CCr (mL/min)	84.25 ± 40.55	95.27 ± 42.38		*P* > 0.05
Albuminuria (mg/day)	305.20 ± 946.28	110.70 ± 238.78		*P* > 0.05
C-Peptide (ng/mL)	2.21 ± 1.77	2.17 ± 1.23		*P* > 0.05
PBG (mg/dL)	171.56 ± 76.20	202.72 ± 99.52	86.87 ± 8.65	(b-c) *P* < 0.05, (a-c) *P* < 0.05
Creatinine (mg/dL)	1.01 ± 0.25	0.85 ± 0.18	0.82 ± 0.15	(a-b) *P* < 0.05, (a-c) *P* < 0.05
Triglyceride (mg/dL)	188.68 ± 109.11	185.44 ± 101.35	141.19 ± 78.74	(a-c) *P* < 0.05, (b-c) *P* < 0.05
HDL (mg/dL)	50.80 ± 13.95	49.74 ± 10.31	54.20 ± 13.18	*P* > 0.05
T. cholesterol (mg/dL)	181.26 ± 46.90	189.16 ± 43.81	203.38 ± 33.94	*P* > 0.05
LDL (mg/dL)	113.40 ± 36.89	121.50 ± 34.24	137.58 ± 32.56	(c-a) *P* < 0.01, (c-b) *P* < 0.05
Sedimentation (mm/hr)	25.38 ± 21.88	18.60 ± 15.35	12.51 ± 8.31	(a-c) *P* < 0.05
CRP (mg/L)	7.71 ± 9.21	6.57 ± 11.42	2.36 ± 1.85	(a-c) *P* < 0.05
Fibrinogen (mg/dL)	386.66 ± 91.74	370.32 ± 68.31	344.51 ± 55.69	(a-c) *P* < 0.05
Homocysteine (umol/L)	11.78 ± 3.85	11.38 ± 3.86	10.58 ± 3.59	*P* > 0.05
B12 (pg/mL)	537.84 ± 381.73	449.14 ± 309.35	421.03 ± 274.66	*P* > 0.05
Folic acid (ng/mL)	9.27 ± 3.57	9.51 ± 3.58	9.93 ± 8.14	*P* > 0.05
Car.Int-R (mm)	0.96 ± 0.23	0.89 ± 0.24	0.78 ± 0.19	(a-c) *P* < 0.05
Car.Int-L (mm)	0.93 ± 0.18	0.93 ± 0.28	0.79 ± 0.19	(a-c) *P* < 0.05, (b-c) *P* < 0.05
ADMA (umol/L)	0.52 ± 0.23	0.39 ± 0.16	0.32 ± 0.13	(a-b) *P* < 0.05, (a-c) *P* < 0.05

BMI: body mass index; SBP: systolic blood pressure; DBP: diastolic blood pressure; D. of diabetes: duration of diabetes.

**Table 2:** Comparison of the common parameters in all diabetic patients and the control group.

	DM	Control	*P* values Significance
Age (years)	59.41 ± 8.81	55.00 ± 7.34	<0.05
Creatinine (mg/dL)	0.93 ± 0.23	0.82 ± 0.16	<0.05
Triglyceride (mg/dL)	187.65 ± 105.15	140.78 ± 77.49	<0.01
T. cholesterol (mg/dL)	185.78 ± 45.18	201.03 ± 35.95	<0.05
HDL (mg/dL)	50.36 ± 12.24	53.79 ± 13.17	>0.05
LDL (mg/dL)	117.91 ± 35.51	135.50 ± 34.13	<0.01
Sedimentation (mm/hr)	21.96 ± 19.21	12.87 ± 8.42	<0.01
hsCRP (mg/L)	7.21 ± 10.37	2.29 ± 1.86	<0.01
Fibrinogen (mg/dL)	377.86 ± 81.06	347.50 ± 57.32	<0.01
Homocysteine (umol/L)	11.58 ± 3.86	10.61 ± 3.53	>0.05
Car.Int-R. (mm)	0.92 ± 0.22	0.80 ± 0.22	<0.01
Car.Int-L (mm)	0.93 ± 0.23	0.80 ± 0.20	<0.01
ADMA (umol/L)	0.45 ± 0.21	0.32 ± 0.13	<0.001

**Table 3:** Correlations between ADMA and creatinine, sedimentation, hsCRP, fibrinogen, and homocysteine in all groups.

	ADMA	
Creatinine (mg/dL)	*r* = 0.173	*P* < 0.05
Sedimentation (mm/hr)	*r* = 0.059	*P* > 0.05
hsCRP (mg/L)	*r* = 0.098	*P* > 0.05
Fibrinogen (mg/dL)	*r* = 0.057	*P* > 0.05
Homocysteine (umol/L)	*r* = 0.069	*P* > 0.05

**Table 4:** Correlations between ADMA and creatinine, sedimentation, hsCRP, fibrinogen, and homocysteine in MVD group.

	ADMA	
Creatinine (mg/dL)	*r* = 0.024	*P* > 0.05
Sedimentation (mm/hr)	*r* = 0.00	*P* > 0.05
hsCRP (mg/L)	*r* = 0.217	*P* > 0.05
Fibrinogen (mg/dL)	*r* = 0.269	*P* > 0.05
Homocysteine (umol/L)	*r* = 0.189	*P* > 0.05

**Table 5:** Multiple regression analysis results in MVD group.

Independent predictors	*β*	*P*
Albuminuria (mg/day)	−0.322	0.21
Triglyceride (mg/dL)	0.254	0.63
Homocysteine (umol/L)	0.403	**0.002**
Car.Int-R (mm)	−0.221	0.81
